# Physical fitness status and influencing factors among college students aged 19 ~ 22 in Shandong province, China: a cross-sectional study

**DOI:** 10.1186/s12889-025-22350-x

**Published:** 2025-06-05

**Authors:** Zhihao Huang, Wance Wang, Kai Huo, Yongming Wang, Kunzong Tian

**Affiliations:** 1 School of Big Data and Fundamental Sciences, Shandong Institute of Petroleum and Chemical Technology, Dongying, China; 2 School of Physical Education and Health, East China Jiaotong University, Nanchang, China; 3 Department of Student Affairs, Shandong Institute of Petroleum and Chemical Technology, Dongying, China

**Keywords:** College students, Physical fitness, Influencing factors, China

## Abstract

**Background:**

College students physical fitness is not just an indicator of current health but also a predictor of future well-being. The rapid socioeconomic changes and significant lifestyle shifts occurring in China, akin to global trends, underscore the need for a comprehensive understanding of college students physical fitness.

**Methods:**

Our research focused on delineating the state of physical fitness among college students in Shandong Province and identifying the factors influencing it. Univariate chi-squared test was initially performed on all independent variables. Subsequently, statistically significant variables were included in multivariate logistic regression analysis to identify the determinants of physical fitness among college students. These findings were intended to inform targeted health interventions for this critical age group.

**Results:**

From September to November 2024, a large-scale survey was conducted on 176,373 college students aged 19 to 22 years in Shandong Province, using a stratified random cluster sampling method. The overall physical fitness qualified rate was 92.03%, with female students slightly outperforming males. Urban students had a higher qualified rate than rural students. After adjusting for gender and household registration, multivariable logistic regression analysis identified key factors influencing physical fitness. Parental engagement in physical exercise and support for students’ participation were associated with lower odds of failing fitness tests. Frequent exercise, long exercise duration, and moderate to high intensity activity were linked to better fitness outcome. Sufficient sleep and regular breakfast consumption also decreased the likelihood of failing the physical fitness tests. In contrast, excessive screen time, smoking, frequent alcohol consumption, and high fast food intake were associated with increased odds of failing physical fitness tests.

**Conclusion:**

These findings highlighted the significant role of lifestyle factors, including parental influence, exercise frequency, duration and intensity, sleep duration, screen time, smoking, alcohol consumption, breakfast consumption habit, and fast food intake, in determining college students’ physical fitness test outcomes.

**Supplementary Information:**

The online version contains supplementary material available at 10.1186/s12889-025-22350-x.

## Background

Physical fitness is a comprehensive indicator of health, reflecting the functional status of multiple body systems, including skeletomuscular, cardiorespiratory, hematocirculatory, psychoneurological, and endocrine-metabolic systems [1]. Assessing physical fitness serves as an indirect evaluation of these systems and is widely recognized as a critical health marker [2]. Empirical evidence highlighted cardiorespiratory capacity and muscular strength as key predictors of chronic diseases such as cardiovascular disease, diabetes, and hypertension [3–5]. The risks associated with poor fitness emerge as early as adolescence [6–9], with robust cardiorespiratory fitness in youth conferring long-term health benefits [10, 11]. In 2016, the American Heart Association classified cardiorespiratory endurance as a vital health metric alongside pulse rate and blood pressure [12]. Obesity in childhood and adolescence is strongly linked to chronic metabolic diseases, including hypertension, dyslipidemia, insulin resistance, and psychosocial complications [13–16]. A significant body of research underscored the positive correlation between physical fitness and overall health in youth [17], prompting global initiatives to enhance fitness levels. In the United States, the Cooper Institute’s longitudinal studies led to the NFL PLAY 60 FitnessGram Project, benefiting millions of children [18]. Europe has also undertaken large-scale initiatives such as the Identification and Prevention of Dietary- and Lifestyle-Induced Health Effects in Children and Infants study, conducted across eight countries [19], and the Healthy Lifestyle in Europe by Nutrition in Adolescence study, which assessed adolescent nutrition and lifestyle across ten cities [20]. China has prioritized youth physical fitness, aligning with the “Healthy China 2030” blueprint, which aimed for an excellence rate exceeding 25% in national student fitness assessments by 2030 [21]. This study examines 2024 physical fitness data of college students aged 19 ~ 22 in Shandong Province, contributing to regional public health strategies and aligning with the United Nations’ Sustainable Development Goals for 2030, particularly Goal 3, which advocates for universal health and well-being [22]. Specifically, we hypothesize that factors such as parental influence, exercise habit, sleep duration, screen time, smoking, alcohol consumption, and dietary habit are significant determinants of physical fitness among this age group. Addressing youth health determinants is essential for fostering a healthier generation capable of supporting sustainable development.

## Materials and methods

### Design and participants

This study adhered to the Strengthening the Reporting of Observational Studies in Epidemiology guidelines [23]. A stratified random cluster sampling method was used to recruit participants from 58 universities and colleges across 16 cities in Shandong Province, encompassing individuals aged 19 to 22 years. Data collection occurred from September to November 2024. Stratification was conducted based on academic year, with entire classes randomly selected as sampling units. The initial survey included 180,892 participants. This meticulous sampling methodology not only ensured accurate representation across different grade levels but also affirmed that the selected subgroups reflected the broader educational landscape of Shandong Province. The primary objective of employing this approach was to minimize potential biases and construct a sample that, while diverse, maintained methodological rigor and precision.

Exclusion criteria were applied to eliminate students with severe organ diseases, physical disabilities, or deformities, resulting in a final sample of 176,373 students who completed both physical fitness tests as well as a comprehensive questionnaire, achieving a response rate of 97.50%. Among the valid respondents, 49.50% (87,302 individuals) were male, and 50.50% (89,071 individuals) were female. The age distribution was as follows: 36.20% (63,847 individuals) were 19 years old, 29.29% (51,659 individuals) were 20 years old, 21.81% (38,467 individuals) were 21 years old, and 12.70% (22,400 individuals) were 22 years old. The mean age of participants was 20.11 ± 1.037 years.

### Assessments of physical fitness and implementation of questionnaire survey

Physical fitness assessments were conducted with precision following the “National Student Physical Health Standard (Revised 2014)” guidelines [24], endorsed by the Ministry of Education. The testing sequences were systematically arranged, beginning with measurements of height (centimeter) and weight (kilogram), followed by the evaluation of vital capacity (milliliter). Subsequent assessments included the sit-and-reach test (centimeter), pull-ups for boys (repetition) and 1-minute sit-ups for girls (repetition), standing long jump (centimeter), 50-meter sprint (second), and, finally, the 1,000-meter run for boys (second) or 800-meter run for girls (second). A composite score of ≥ 60 was established as the benchmark for satisfactory physical fitness proficiency. This score represented the aggregate of individual metrics, each assigned a specific weight. The distribution of these weights was as follows: body mass index (calculated as weight in kilogram divided by the square of height in meter) accounted for 15%, vital capacity for 15%, sit-and-reach for 10%, pull-ups for boys or 1-minute sit-ups for girls for 10%, standing long jump for 10%, 50-meter sprint for 20%, and 1,000-meter run for boys or 800-meter run for girls for 20%. The questionnaires, conducted under strict assurances of respondent anonymity, explored several key domains: Demographic characteristics: gender, age, household registration, family annual income (yuan), paternal education level, maternal education level; Home environments: whether parents liked physical exercise, whether parents support your participation in physical exercise; Physical exercise status: number of physical exercise session per week (count), duration of each physical exercise session (hour), intensity of physical exercise; Lifestyle factors: sleep duration per day (hour), screen duration per day (hour), times of smoking per week, times of drinking per week; Dietary behaviors: times of breakfast intake per week, times of meat intake per week, times of vegetable intake per week, times of fruit intake per week, times of eggs intake per week, times of milk intake per week, times of fast food consumption per week (Supplementary Tables 1 and Supplementary Table 2).

All testers and investigators involved in this study underwent standardized training to ensure proficiency in conducting physical fitness assessments and field epidemiological investigation techniques. Prior to the commencement of the survey, students were provided with a comprehensive briefing outlining the study’s objectives and significance. All physical fitness tests, except for the 1,000-meter test for boys and the 800-meter test for girls, were conducted three times, with the best result taken. Following the completion of the physical fitness tests, questionnaires were distributed, allowing students one day to consult with their parents before completing them. The questionnaires were redistributed two weeks later, with incomplete, illegible, or inconsistent responses across both rounds excluded. The completed questionnaires were then promptly collected, systematically organized, and entered into the database for subsequent data analysis. This study received ethical approval from the Ethics Committee of Shandong Institute of Petroleum and Chemical Technology (registration number: KY-2024-032). All procedures adhered strictly to relevant guidelines and regulations. Additionally, informed consents were obtained from all participants prior to their involvement in the study.

### Statistical analysis

A comprehensive database was constructed using EpiData 3.1 (EpiData Association, Odense, the Kingdom of Denmark), and subsequent data analyses were conducted using Statistical Product and Service Solutions 27.0 (International Business Machines Corporation, New York, the United States of America). Continuous variables were reported as means with standard deviations, while categorical variables were presented as frequencies with corresponding percentage (%). For univariable analysis, the chi-squared test was employed to identify significant associations. Variables demonstrating statistical significance (*P* < 0.05) in the univariable analysis were subsequently included in the multivariable logistic regression analysis [25]. In the multivariable phase, logistic regression was performed, with results expressed as odds ratio (OR) accompanied by 95% confidence interval (CI) to provide precise estimates [26]. To evaluate the reliability of the findings, the model’s fit was assessed using the Hosmer-Lemeshow goodness-of-fit test. This rigorous methodological approach ensured the robustness of the model, highlighting the integrity and precision of the analytical framework and adhering to the principles of statistical rigor.

## Results

### Qualified rates of physical fitness tests among diverse demographic groups of college students

Based on the 2024 survey data of college students aged 19 ~ 22 in Shandong Province, the overall qualified rate for physical fitness was 92.03%. Notably, the study revealed a slightly higher qualified rate among female students (92.18%) compared to their male counterparts (91.88%), with the difference reaching statistical significance (*P* < 0.05). Furthermore, students with an urban household registration demonstrated a significantly higher qualified rate (92.20%) than those with a rural household registration (91.70%) (*P* < 0.05) (Table 1).

**Table 1:** Qualified rate according to demographic characteristics of college students aged 19 ~ 22 in Shandong Province who participated in the 2024 physical fitness test and questionnaire survey

Demographic characteristics	Number of participants who passed the physical fitness test(%)	Number of participants who failed the physical fitness test(%)	χ^2^	*P*
Gender			5.428	0.020
Male	80,214(91.88)	7,088(8.12)		
Female	82,107(92.18)	6,964(7.82)		
Age (year)			1.761	0.623
19	58,690(91.92)	5,157(8.08)		
20	47,577(92.10)	4,082(7.90)		
21	35,436(92.12)	3,031(7.88)		
22	20,618(92.04)	1,782(7.96)		
Household registration			13.430	0.000
Rural	54,465(91.70)	4,929(8.30)		
Urban	107,856(92.20)	9,123(7.80)		
Family annual income (yuan)			3.795	0.284
≤ 100,000	55,401(91.86)	4,906(8.14)		
100,001 ~ 200,000	72,911(92.09)	6,261(7.91)		
200,001 ~ 300,000	21,146(92.17)	1,796(7.83)		
≥ 300,000	12,863(92.19)	1,089(7.81)		
Paternal education level			0.607	0.738
Junior high school or below	29,737(91.93)	2,609(8.07)		
Senior high school	48,162(92.03)	4,172(7.97)		
Above senior high school	84,422(92.07)	7,271(7.93)		
Maternal education level			0.667	0.716
Junior high school or below	31,666(91.93)	2,779(8.07)		
Senior high school	44,247(92.03)	3,832(7.97)		
Above senior high school	86,408(92.07)	7,441(7.93)		

### Univariable analysis of qualified rates in college students’ physical fitness tests

The univariable analysis revealed that several factors were significantly associated with the qualified rate of physical fitness among college students aged 19 ~ 22. These factors included whether parents like physical exercise, whether parents support your participation in physical exercise, number of physical exercise sessions per week, duration of each physical exercise session (hour), intensity of physical exercise, sleep duration per day (hour), screen duration per day (hour), times of smoking per week, times of drinking per week, times of breakfast intake per week, times of fast food consumption per week (*P* < 0.05) (Table 2).

### Multivariable logistic regression analysis of factors influencing the qualified rate in college students’ physical fitness tests

In this study, students’ physical fitness test results—classified dichotomously as 0 for “qualified” and 1 for “unqualified”—were employed as the dependent variable. Guided by strict adherence to established inclusion and exclusion criteria and a significance level set at *P* = 0.05, a comprehensive multivariable logistic regression analysis was conducted. This analysis incorporated 13 independent variables, encompassing a range of gender, household registration, whether parents like physical exercise, whether parents support your participation in physical exercise, number of physical exercise sessions per week, duration of each physical exercise session (hour), intensity of physical exercise, sleep duration per day (hour), screen duration per day (hour), times of smoking per week, times of drinking per week, times of breakfast intake per week, times of fast food consumption per week. However, the results of the Hosmer-Lemeshow test indicated that the logistic regression model did not achieve an optimal fit (*P* < 0.05), implying potential issues with the model’s adequacy. A more detailed examination of the multivariable logistic regression analysis revealed that, after adjusting for gender and household registration characteristics, college students exhibited significantly lower odds of failing physical fitness tests under several conditions. Specifically, lower odds were observed among college students with one parent or both parents who engaged in physical exercise, compared to those whose parents did not. Similarly, college students whose parents supported their participation in physical exercise had reduced odds of failure compared to those whose parents did not provide support. Participation in physical exercise 3 ~ 5 times per week or more than 5 times per week was associated with lower odds of failure compared to exercising fewer than 3 times per week. Additionally, exercising for 0.5 ~ 1 h per session or more than 1 h per session was linked to reduced odds compared to sessions of less than 0.5 h. Engagement in moderate intensity and high intensity physical exercise was associated with lower odds of failing compared to low intensity exercise. Adequate sleep duration of 6 ~ 8 h per day or more than 8 h per day was similarly associated with lower odds of failure compared to sleeping less than 6 h. Regular breakfast consumption also played a significant role, with reduced odds of failure observed in college students consuming breakfast 1 ~ 2 times per week, 3 ~ 6 times per week, or daily compared to those who did not consume breakfast regularly. Conversely, an increased likelihood of failing physical fitness tests was observed among college students who spent 1 ~ 3 h per day on screens or more than 3 h per day, compared to those who spent less than 1 h per day. College students who smoked 2 ~ 4 times or more than 4 times had higher odds of failing compared to those who never smoked. Similarly, students who drank alcohol 2 ~ 4 times or more than 4 times had greater odds of failing compared to those who drank less than 2 times. Additionally, the consumption of fast food 2 ~ 3 times per week or more than 3 times per week was associated with increased odds of failure compared to consuming fast food less than 2 times per week (Table 3).

**Table 2:** Univariable analysis of qualified rate among college students aged 19 ~ 22 in Shandong Province who participated in the 2024 physical fitness test and questionnaire survey

Independent variables	Number of participants who passed the physical fitness test(%)	Number of participants who failed the physical fitness test(%)	χ^2^	*P*
Whether parents like physical exercise			21.546	0.000
Neither side of the parents likes	54,191(91.61)	4,962(8.39)		
One side of the parents likes	73,839(92.24)	6,212(7.76)		
Both sides of the parents like	34,291(92.26)	2,878(7.74)		
Whether parents support your participation in physical exercise			7.349	0.007
Nonsupporting	11,436(91.40)	1,076(8.60)		
Supporting	150,885(92.08)	12,976(7.92)		
Number of physical exercise sessions per week			30.595	0.000
< 3	35,006(91.36)	3,312(8.64)		
3 ~ 5	91,570(92.21)	7,737(7.79)		
> 5	35,745(92.25)	3,003(7.75)		
Duration of each physical exercise session (hour)			13.981	0.000
< 0.5	35,601(91.65)	3,242(8.35)		
0.5 ~ 1	109,134(92.08)	9,387(7.92)		
> 1	17,586(92.51)	1,423(7.49)		
Intensity of physical exercise			6.651	0.036
Low intensity	13,726(91.56)	1,266(8.44)		
Moderate intensity	140,342(92.10)	12,042(7.90)		
High intensity	8,253(91.73)	744(8.27)		
Sleep duration per day (hour)			17.409	0.000
< 6	11,908(91.14)	1,157(8.86)		
6 ~ 8	85,914(92.02)	7,453(7.98)		
> 8	64,499(92.22)	5,442(7.78)		
Screen duration per day (hour)			6.399	0.041
< 1	12,058(92.29)	1,008(7.71)		
1 ~ 3	102,274(92.12)	8,752(7.88)		
> 3	47,989(91.79)	4,292(8.21)		
Times of smoking per week			61.224	0.000
Never	119,870(92.20)	10,140(7.80)		
1 ~ 4	27,941(92.16)	2,378(7.84)		
> 4	14,510(90.44)	1,534(9.56)		
Times of drinking per week			50.774	0.000
< 2	84,798(92.25)	7,122(7.75)		
2 ~ 4	53,002(92.19)	4,491(7.81)		
> 4	24,521(90.95)	2,439(9.05)		
Times of breakfast intake per week			40.720	0.000
Never	3,689(89.98)	411(10.02)		
1 ~ 2	17,532(91.62)	1,603(8.38)		
3 ~ 6	22,465(91.63)	2,052(8.37)		
Everyday	118,635(92.24)	9,986(7.76)		
Times of meat intake per week			0.106	0.948
< 3	76,119(92.02)	6,600(7.98)		
3 ~ 4	59,242(92.03)	5,133(7.97)		
> 4	26,960(92.08)	2,319(7.92)		
Times of vegetable intake per week			5.668	0.059
< 4	13,747(91.62)	1,257(8.38)		
4 ~ 5	40,854(91.92)	3,591(8.08)		
> 5	107,720(92.13)	9,204(7.87)		
Times of fruit intake per week			1.843	0.398
< 4	37,431(91.87)	3,311(8.13)		
4 ~ 5	75,191(92.08)	6,470(7.92)		
> 5	49,699(92.09)	4,271(7.91)		
Times of eggs intake per week			2.002	0.368
< 4	20,084(91.81)	1,791(8.19)		
4 ~ 5	99,019(92.04)	8,567(7.96)		
> 5	43,218(92.13)	3,694(7.87)		
Times of milk intake per week			3.515	0.172
< 4	24,759(91.75)	2,226(8.25)		
4 ~ 5	101,658(92.07)	8,752(7.93)		
> 5	35,904(92.11)	3,074(7.89)		
Times of fast food consumption per week			6.326	0.042
< 2	111,322(92.14)	9,494(7.86)		
2 ~ 3	37,085(91.82)	3,304(8.18)		
> 3	13,914(91.73)	1,254(8.27)		

**Table 3:** Multivariable analysis of qualified rate among college students aged 19 ~ 22 in Shandong Province who participated in 2024 physical fitness test and questionnaire survey

Independent variables	OR(95%CI)	*P*
Whether parents like physical exercise		
Neither side of the parents likes	1	
One side of the parents likes	0.921(0.854 ~ 0.993)	0.031
Both sides of the parents like	0.711(0.607 ~ 0.833)	0.000
Whether parents support your participation in physical exercise		
Nonsupporting	1	
Supporting	0.916(0.858 ~ 0.978)	0.008
Number of physical exercise sessions per week		
< 3	1	
3 ~ 5	0.906(0.828 ~ 0.992)	0.032
> 5	0.810(0.695 ~ 0.945)	0.007
Duration of each physical exercise session (hour)		
< 0.5	1	
0.5 ~ 1	0.912(0.873 ~ 0.954)	0.000
> 1	0.516(0.448 ~ 0.595)	0.000
Intensity of physical exercise		
Low intensity	1	
Moderate intensity	0.760(0.660 ~ 0.876)	0.000
High intensity	0.793(0.665 ~ 0.946)	0.010
Sleep duration per day (hour)		
< 6	1	
6 ~ 8	0.907(0.848 ~ 0.970)	0.005
> 8	0.906(0.838 ~ 0.979)	0.013
Screen duration per day (hour)		
< 1	1	
1 ~ 3	1.753(1.521 ~ 2.021)	0.000
> 3	2.086(1.791 ~ 2.431)	0.000
Times of smoking per week		
Never	1	
1 ~ 4	1.206(1.076 ~ 1.353)	0.001
> 4	1.147(1.023 ~ 1.285)	0.019
Times of drinking per week		
< 2	1	
2 ~ 4	1.078(1.018 ~ 1.140)	0.010
> 4	1.152(1.032 ~ 1.286)	0.012
Times of breakfast intake per week		
Never	1	
1 ~ 2	0.694(0.610 ~ 0.788)	0.000
3 ~ 6	0.663(0.567 ~ 0.775)	0.000
Everyday	0.661(0.552 ~ 0.792)	0.000
Times of fast food consumption weekly		
< 2	1	
2 **~** 3	1.124(1.075 ~ 1.175)	0.000
> 3	1.898(1.638 ~ 2.198)	0.000

## Discussion

This study underscored a significant relationship between parental preferences and the promotion of physical activity, which markedly influenced the rate of successful physical fitness test outcomes among college students. The familial environment exerts a substantial impact on the developmental trajectory of a child. Behaviors established during early life stages, shaped by educational and environmental exposures, tend to become ingrained, resembling intrinsic traits that persist throughout an individual’s lifetime. In a foundational study, researchers investigated the determinants influencing children’s physical activity behaviors through a “lifecycle perspective” [27]. The findings revealed that the formative years, particularly up to the age of 16, played a critical role in molding physical activity habits. The conditions and experiences during this pivotal period emerged as key factors driving disparities in exercise engagement. Notably, the study highlighted the enduring influence of the family environment, identifying it as a persistent determinant that is difficult to modify. Enhancing a family’s sports-oriented atmosphere has been shown to have a positive effect on adolescents’ physical health [28]. Parental endorsement of physical activity serves as a crucial predictor of adolescents’ participation in such activities. The perception of parental support often motivates children to engage more enthusiastically in physical exercise. Additionally, increased parental involvement in physical activities can act as a powerful catalyst, encouraging adolescents to allocate more time to similar pursuits, thereby promoting better physical health outcomes.

This study highlighted the significant impact of exercise behaviors—including frequency, duration, and intensity—on the success rate of physical fitness assessments among college students. A well-established understanding of physical activity, combined with consistent participation, has been shown to be a critical determinant for the comprehensive improvement of adolescent physical fitness [29]. Engaging in regular physical exercise leads to multifaceted enhancements in various physiological attributes, such as endurance, strength, speed, and flexibility.Aerobic exercises, including activities like running, swimming, and cycling, have been demonstrated to enhance cardiorespiratory function, thereby increasing stamina and resilience in middle school students [30]. Concurrently, anaerobic exercises, such as pull-ups and push-ups, contribute to greater muscle strength and promote healthy skeletal development [31]. Additionally, high-intensity anaerobic exercises, such as sprinting, improve speed and explosive power in adolescents [32]. Flexibility training, represented by activities like gymnastics and yoga, expands joint mobility, reduces muscle stiffness, and enhances overall physical pliability and agility [33]. The influence of physical activity on adolescent health and fitness is both profound and invaluable. Recognizing adolescence as a critical period for psychophysical development, exercise not only facilitates growth and maturation [34] but also enhances coordination skills [35], fosters teamwork [36], and cultivates a positive outlook on life [37]. However, one of the most notable findings of this study is the superior effectiveness of moderate intensity exercise in promoting physical fitness among college students In contrast, the shift to high intensity exercise did not yield proportional improvements. These findings suggest that while exercise intensity is an essential factor influencing adolescent fitness, indiscriminately increasing exercise intensity may not lead to the anticipated benefits and could potentially result in negative physiological and psychological consequences, thereby hindering optimal adolescent development. The research underscored the importance of tailored exercise regimens that emphasized moderate intensity activities for sustainable physical fitness improvements in youth.

This investigation’s findings emphasized the critical role of sleep duration in influencing the qualified rate of physical fitness tests among college students. Sleep, an essential physiological process, holds significant importance, particularly for adolescents due to their ongoing physical growth and development. Adequate sleep is therefore indispensable [38]. Notably, during nocturnal rest, there is an increased secretion of growth hormone [39], which is vital for adolescent physical development, especially in bone and muscle growth. Moreover, sleep is fundamental for energy recovery, which is crucial for adolescents engaged in prolonged sports and physical activities. During sleep, the body conducts complex self-reparative processes that facilitate energy restoration [40]. Insufficient sleep can lead to significant physical fatigue, adversely affecting daily physiological functions. Additionally, sleep plays a pivotal role in maintaining neuromuscular coordination and reflex speed. Sleep deprivation or poor-quality sleep can disrupt neuromuscular connections, resulting in slower reaction times [41]. Furthermore, sleep is essential for memory consolidation and the acquisition of new skills [42]. Inadequate sleep can impair the learning of new athletic skills, such as precise technical movements. Finally, sufficient sleep is crucial for maintaining immune function [43]. Adequate sleep strengthens the immune system in adolescents, enhancing their resilience against various illnesses. These findings collectively underscored the necessity of ensuring sufficient sleep for adolescents to support their physical fitness, coordination, learning capacity, and overall health.

This study highlighted the significant implications of sedentary behaviors, particularly those characterized by prolonged screen exposure, on the physical fitness test performance of college students. As society progresses, the academic demands placed on adolescents have intensified. Simultaneously, the pervasive presence of electronic devices has seamlessly integrated into daily life, fostering an increasingly sedentary lifestyle among college students and consequently extending their periods of inactivity. These sedentary tendencies are now emerging as a growing public health concern [44]. Fundamentally, sedentary behaviors have been shown to contribute to a reduction in bone mineral density in adolescents [45] and promote muscular atrophy [46]. Additionally, prolonged periods of sitting, combined with poor posture, may lead to orthopedic misalignments such as scoliosis [47]. These postural deviations not only impair physical appearance but also create potential for long-term health issues. Moreover, the sedentary lifestyle is often associated with lower energy expenditure. When caloric intake is not appropriately adjusted, this imbalance can lead to weight gain, thereby increasing the risk of overweight and obesity [48]. Finally, sedentary postures are linked to negative effects on cardiovascular health. Chronic engagement in sedentary routines places undue strain on the cardiovascular system, ultimately elevating the risk of chronic diseases, including cardiovascular disorders [49]. The increasing prevalence of sedentary behaviors among college students, driven by modern academic and technological shifts, presents a multifaceted challenge to public health. Addressing this issue requires a comprehensive approach, incorporating educational programs, lifestyle modifications, and policy interventions to reduce sedentary time.

This study highlighted the detrimental effects of prolonged smoking on the physical fitness performance of college students. Notably, adolescence is identified as a critical period during which individuals exhibit heightened vulnerability to the harmful impacts of tobacco exposure [50]. Existing literature indicated that smoking during adolescence could adversely affect asthma, lung function, and lung development, with repercussions that extended into later stages of growth [51]. These effects can result in diminished endurance levels, which are crucial for overall physical performance. Accumulating evidence indicated that smoking may also compromise the immune system [52], thereby heightening susceptibility to infectious agents. Moreover, smoking has been linked to an increased risk of cardiovascular conditions [53], including hypertension [54] and disturbances in heart rate variability [55]. Additionally, the potential adverse impacts on skeletal health [56] and muscular development [57] are concerning, as they may further impair physical performance. Smoking during adolescence is also associated with potential disruptions in neurodevelopmental trajectories, adding to the array of long-term health risks. The pervasive effects of smoking extend beyond physical health, contributing to cognitive impairments and learning difficulties. These findings reinforced the urgency of implementing protective measures for adolescents to shield them from the detrimental consequences of tobacco exposure during such a vital phase of development.

This study found that alcohol consumption affected the pass rate of physical fitness tests among university students. Firstly, alcohol disrupts whole-body metabolism [58], particularly the normal metabolic activity of the liver. The liver is responsible for processing alcohol [59], but when overloaded, it can impair the metabolism of fats and sugars, thereby weakening endurance and speed performance. Secondly, alcohol inhibits protein synthesis [60], affecting muscle growth and repair, leading to decreased muscle strength and reduced recovery capacity due to dehydration. Additionally, alcohol intake interferes with the normal regulation of heart rate and blood pressure [61], decreasing aerobic capacity and cardiovascular endurance. Chronic alcohol consumption can also result in excessive caloric intake [62], increasing body fat percentage and complicating weight control, which negatively impacts flexibility performance. Furthermore, excessive alcohol consumption suppresses immune function [63], making the body more susceptible to infections and inflammation, thereby reducing overall health and further impairing physical fitness and recovery. Alcohol consumption poses multiple adverse effects on various aspects of physical fitness. The cumulative impact of alcohol on metabolism, muscle function, cardiovascular regulation, and immune response underscores the importance of promoting awareness and educational interventions to mitigate alcohol-related health risks among college students.

This study identified significant correlations between dietary behaviors—specifically, the regularity of breakfast consumption and the frequency of fast food intake—and the performance outcomes on physical fitness assessments among adolescents. Dietary habits are pivotal determinants of an individual’s health trajectory [64], particularly during adolescence, a phase marked by critical growth and developmental milestones [65]. The importance of breakfast as a fundamental component of daily nutritional routines is well-established [66]. As the first meal of the day, breakfast replenishes energy reserves depleted overnight, stimulates metabolic processes, and enhances cognitive functions such as memory and creativity, potentially contributing to improved exercise performance [67]. Given the heightened metabolic and developmental demands of adolescence, habitual omission of breakfast can lead to nutritional deficiencies, adversely affecting overall growth and development [68] Conversely, while fast food is often favored for its convenience—particularly by adolescents with limited time—it typically presents substantial nutritional limitations. Characterized by excessive calories, sugars, salts, and fats, fast food frequently lacks essential proteins, vitamins, minerals, and dietary fiber that are necessary for balanced nutrition [69]. Dependence on such nutritionally deficient foods not only raises the risk of malnutrition [70] but can also contribute to weight gain, thereby increasing the likelihood of obesity [71]. These findings underscored the critical role of dietary practices in shaping adolescent health outcomes, emphasizing the need for educational interventions and policies that promote healthy eating habits to support physical fitness and holistic development during this pivotal stage.

## Conclusion

Families, educational institutions, and broader society must adopt a coordinated approach to enhance the physical health of college students, promoting balanced and holistic development. Parental involvement, both through active participation and as behavioral role models, plays a critical role in fostering healthy lifestyle habits among students. Educational institutions, in collaboration with families and societal organizations, should advocate for and facilitate student engagement in physical activities, supported by the provision of appropriate sports facilities and an enabling environment. College students should strive to balance academic responsibilities with relaxation, ensuring sufficient sleep and minimizing prolonged sedentary periods. Additionally, refraining from smoking and alcohol consumption, along with maintaining a balanced diet that meets students’ nutritional requirements, is crucial. By implementing these strategies, the physical well-being of college students can be strengthened, supporting their overall development. Despite the significant findings of this study, it is essential to interpret the results in light of certain limitations. The use of the Hosmer-Lemeshow test indicated that the logistic regression model may not have exhibited an ideal fit (*P* < 0.05), suggesting potential discrepancies in accurately predicting the relationship between variables and physical fitness tests, which could affect the validity of the findings. Another notable limitation involves potential measurement errors. The reliability of self-reported data, especially from questionnaires, remained a concern, as recall biases may impact students’ recollections of activities and social desirability bias could compromise the accuracy of their responses. The cross-sectional design of the study offered only a snapshot in time, limiting the ability to infer causal relationships or track changes over time. Additionally, uncontrolled confounders, such as genetic predispositions, environmental factors like air quality, and temporary physical conditions on the day of testing, were not accounted for in the study. Finally, the geographical focus on Shandong Province may restrict the generalizability of the findings to regions or countries with different cultural, socioeconomic, or environmental contexts. On the other hand, the study’s large sample size of 176,373 college students aged 19 to 22 years significantly enhanced the reliability of the findings, and the stratified random cluster sampling method contributed to the representativeness of the sample. By examining a broad range of factors, the study provided a comprehensive view of the determinants of physical fitness. The use of multivariable logistic regression analysis further strengthened the study by identifying key factors associated with fitness outcomes, offering a nuanced understanding of the variables influencing physical fitness among college students.

## Electronic Supplementary Material

Below is the link to the electronic supplementary material.

Supplementary Material 1

Supplementary Material 2

## Data Availability

Data is provided within the manuscript or supplementary information files.

## Abbreviations

OR: Odds ratio
CI: Confidence interval

## References

[1] Tomkinson GR, Carver KD, Atkinson F, Daniell ND, Lewis LK, Fitzgerald JS, Lang JJ, Ortega FB. European normative values for physical fitness in children and adolescents aged 9–17 years: results from 2779165 Eurofit performances representing 30 countries. Br J Sports Med. 2018;52(22):1445–56. 10.1136/bjsports-2017-098253. 29191931 10.1136/bjsports-2017-098253

[2] Ortega FB, Ruiz JR, Castillo MJ, Sjöström M. Physical fitness in childhood and adolescence: a powerful marker of health. Int J Obes (Lond). 2008;32(1):1–11. 10.1038/sj.ijo.0803774. 18043605 10.1038/sj.ijo.0803774

[3] Al-Mallah MH, Sakr S, Al-Qunaibet A. Cardiorespiratory fitness and cardiovascular disease prevention: an update. Curr Atheroscler Rep. 2018;20(1):1. 10.1007/s11883-018-0711-4. 29340805 10.1007/s11883-018-0711-4

[4] Elagizi A, Kachur S, Lavie CJ, Carbone S, Pandey A, Ortega FB, Milani RV. An overview and update on obesity and the obesity paradox in cardiovascular diseases. Prog Cardiovasc Dis. 2018;61(2):142–50. 10.1016/j.pcad.2018.07.003. 29981771 10.1016/j.pcad.2018.07.003

[5] Henriksson H, Henriksson P, Tynelius P, Ekstedt M, Berglind D, Labayen I, Ruiz JR, Lavie CJ, Ortega FB. Cardiorespiratory fitness, muscular strength, and obesity in adolescence and later chronic disability due to cardiovascular disease: a cohort study of 1 million men. Eur Heart J. 2020;41(15):1503–10. 10.1093/eurheartj/ehz774. 31710669 10.1093/eurheartj/ehz774 PMC7154806

[6] Timpka S, Petersson IF, Zhou C, Englund M. Muscle strength in adolescent men and risk of cardiovascular disease events and mortality in middle age: a prospective cohort study. BMC Med. 2014;12:62. 10.1186/1741-7015-12-62. 24731728 10.1186/1741-7015-12-62 PMC4006633

[7] Poitras VJ, Gray CE, Borghese MM, Carson V, Chaput JP, Janssen I, Katzmarzyk PT, Pate RR, Connor Gorber S, Kho ME, Sampson M, Tremblay MS. Systematic review of the relationships between objectively measured physical activity and health indicators in school-aged children and youth. Appl Physiol Nutr Metab. 2016;41(6 Suppl 3):S197–239. 10.1139/apnm-2015-0663. 27306431 10.1139/apnm-2015-0663

[8] Henriksson H, Henriksson P, Tynelius P, Ortega FB. Muscular weakness in adolescence is associated with disability 30 years later: a population-based cohort study of 1.2 million men. Br J Sports Med. 2019;53(19):1221–30. 10.1136/bjsports-2017-098723. 29921654 10.1136/bjsports-2017-098723

[9] Henriksson P, Henriksson H, Tynelius P, Berglind D, Löf M, Lee IM, Shiroma EJ, Ortega FB. Fitness and body mass index during adolescence and disability later in life: A cohort study. Ann Intern Med. 2019;170(4):230–9. 10.7326/M18-1861. 30743265 10.7326/M18-1861 PMC6814012

[10] Mintjens S, Menting MD, Daams JG, van Poppel MNM, Roseboom TJ, Gemke RJBJ. Cardiorespiratory fitness in childhood and adolescence affects future cardiovascular risk factors: A systematic review of longitudinal studies. Sports Med. 2018;48(11):2577–605. 10.1007/s40279-018-0974-5. 30144022 10.1007/s40279-018-0974-5 PMC6182463

[11] Smith JJ, Eather N, Morgan PJ, Plotnikoff RC, Faigenbaum AD, Lubans DR. The health benefits of muscular fitness for children and adolescents: a systematic review and meta-analysis. Sports Med. 2014;44(9):1209–23. 10.1007/s40279-014-0196-4. 24788950 10.1007/s40279-014-0196-4

[12] Ross R, Blair SN, Arena R, Church TS, Després JP, Franklin BA, Haskell WL, Kaminsky LA, Levine BD, Lavie CJ, Myers J, Niebauer J, Sallis R, Sawada SS, Sui X, Wisløff U. Importance of assessing cardiorespiratory fitness in clinical practice: A case for fitness as a clinical vital sign: A scientific statement from the American Heart Association. Circulation. 2016;134(24):e653–99. 10.1161/CIR.0000000000000461. 27881567 10.1161/CIR.0000000000000461

[13] Meyer JF, Larsen SB, Blond K, Damsgaard CT, Bjerregaard LG, Baker JL. Associations between body mass index and height during childhood and adolescence and the risk of coronary heart disease in adulthood: A systematic review and meta-analysis. Obes Rev. 2021;22(9):e13276. 10.1111/obr.13276. 33960625 10.1111/obr.13276

[14] Twig G, Reichman B, Afek A, Derazne E, Hamiel U, Furer A, Gershovitz L, Bader T, Cukierman-Yaffe T, Kark JD, Pinhas-Hamiel O. Severe obesity and cardio-metabolic comorbidities: a nationwide study of 2.8 million adolescents. Int J Obes (Lond). 2019;43(7):1391–9. 10.1038/s41366-018-0213-z. 30258119 10.1038/s41366-018-0213-z

[15] Fishman B, Zloof Y, Orr O, Tsur AM, Furer A, Omer Gilon M, Chodick G, Leiba A, Derazne E, Tzur D, Afek A, Grossman E, Twig G. The opposing trends of body mass index and blood pressure during 1977–2020; nationwide registry of 2.8 million male and female adolescents. Cardiovasc Diabetol. 2021;20(1):242. 10.1186/s12933-021-01433-0. 34963457 10.1186/s12933-021-01433-0 PMC8715587

[16] Güngör NK. Overweight and obesity in children and adolescents. J Clin Res Pediatr Endocrinol. 2014;6(3):129–43. 10.4274/Jcrpe.1471. 25241606 10.4274/jcrpe.1471 PMC4293641

[17] Yi X, Fu Y, Burns RD, Bai Y, Zhang P. Body mass index and physical fitness among Chinese adolescents from Shandong Province: a cross-sectional study. BMC Public Health. 2019;19(1):81. 10.1186/s12889-019-6420-2. 30654789 10.1186/s12889-019-6420-2 PMC6337823

[18] Saint-Maurice PF, Bai Y, Welk GJ, Bandelli LN, Allums-Featherston K, Candelaria N. Impact of NFL PLAY 60 Programming on Elementary School Children’s Body Mass Index and Aerobic Capacity: The NFL PLAY 60 FitnessGram Partnership Project. J Sch Health. 2017;87(11):873–81. 10.1111/josh.12561. 29023836 10.1111/josh.12561

[19] De Miguel-Etayo P, Gracia-Marco L, Ortega FB, Intemann T, Foraita R, Lissner L, Oja L, Barba G, Michels N, Tornaritis M, Molnár D, Pitsiladis Y, Ahrens W, Moreno LA, IDEFICS consortium. Physical fitness reference standards in European children: the IDEFICS study. Int J Obes (Lond). 2014;38(Suppl 2):S57–66. 10.1038/ijo.2014.136. 25376221 10.1038/ijo.2014.136

[20] Moreno LA, De Henauw S, González-Gross M, Kersting M, Molnár D, Gottrand F, Barrios L, Sjöström M, Manios Y, Gilbert CC, Leclercq C, Widhalm K, Kafatos A, Marcos A. Design and implementation of the Healthy Lifestyle in Europe by Nutrition in Adolescence Cross-Sectional Study. Int J Obes (Lond). 2008;32(Suppl 5):S4–11. 10.1038/ijo.2008.177. 19011652 10.1038/ijo.2008.177

[21] Dong B, Zou Z, Song Y, Hu P, Luo D, Wen B, Gao D, Wang X, Yang Z, Ma Y, Ma J, Narayan A, Huang X, Tian X, Patton GC. Adolescent Health and Healthy China 2030: A Review. J Adolesc Health. 2020;67(5S):S24–31. 10.1016/j.jadohealth.2020.07.023. 33246530 10.1016/j.jadohealth.2020.07.023

[22] Huang YK, Chang YC. Oral health: The first step to sustainable development goal 3. J Formos Med Assoc. 2022;121(7):1348–50. 10.1016/j.jfma.2021.10.018. 34732302 10.1016/j.jfma.2021.10.018

[23] von Elm E, Altman DG, Egger M, Pocock SJ, Gøtzsche PC, Vandenbroucke JP. The Strengthening the Reporting of Observational Studies in Epidemiology (STROBE) statement: guidelines for reporting observational studies. Lancet. 2007;370(9596):1453–7. 10.1016/S0140-6736(07)61602-X. 18064739 10.1016/S0140-6736(07)61602-X

[24] Zhai X, Ye M, Gu Q, Huang T, Wang K, Chen Z, Fan X. The relationship between physical fitness and academic performance among Chinese college students. J Am Coll Health. 2022;70(2):395–403. 10.1080/07448481.2020.1751643. 32369716 10.1080/07448481.2020.1751643

[25] Bzovsky S, Phillips MR, Guymer RH, Wykoff CC, Thabane L, Bhandari M, Chaudhary V. The clinician’s guide to interpreting a regression analysis. Eye (Lond). 2022;36(9):1715–7. 10.1038/s41433-022-01949-z. 35102247 10.1038/s41433-022-01949-z PMC9391441

[26] Huang Z, Li S, Lu F, Tian K, Peng L. Current situation and factors influencing physical fitness among adolescents aged 12 ~ 15 in Shandong Province, China: A cross-sectional study. Prev Med Rep. 2023;36:102460. 10.1016/j.pmedr.2023.102460. 37927974 10.1016/j.pmedr.2023.102460 PMC10622685

[27] Birchwood D, Roberts K, Pollock G. Explaining differences in sport participation rates among young adults: evidence from the South Caucasus. Eur Phys Educ Rev. 2008;14(3):283–98. 10.1177/1356336X08095667.

[28] Sánchez-Zamorano LM, Solano-González M, Macias-Morales N, Flores-Sánchez G, Galván-Portillo MV, Lazcano-Ponce EC. Perception of parents’ physical activity as a positive model on physical activity of adolescents. Prev Med. 2019;127:105797. 10.1016/j.ypmed.2019.105797. 31404568 10.1016/j.ypmed.2019.105797

[29] Chen W, Hammond-Bennett A, Hypnar A, Mason S. Health-related physical fitness and physical activity in elementary school students. BMC Public Health. 2018;18(1):195. 10.1186/s12889-018-5107-4. 29378563 10.1186/s12889-018-5107-4 PMC5789625

[30] Li H, Xu L. Analysis of cardiopulmonary resistance under different loads in aerobic exercises. Rev Bras Med Esporte. 2022;28(6):624–7. 10.1590/1517-8692202228062022_0078.

[31] Biette N, Tourny-Chollet C, Beuret-Blanquart F. A randomised controlled trial of different leg extension exercises to increase quadriceps muscle strength in female students. Isokinet Exerc Sci. 2004;12(3):209–13. 10.3233/IES-2004-0176.

[32] Markovic G, Jukic I, Milanovic D, Metikos D. Effects of sprint and plyometric training on muscle function and athletic performance. J Strength Cond Res. 2005;21(2):543–9. 10.1519/R-19535.1. 10.1519/R-19535.1 17530960

[33] Zakas A. The effect of stretching duration on the lower-extremity flexibility of adolescent soccer players. J Bodyw Mov Ther. 2005;9(3):220–5. 10.1016/j.jbmt.2004.07.002.

[34] Hallal PC, Victora CG, Azevedo MR, Wells JC. Adolescent physical activity and health: a systematic review. Sports Med. 2006;36(12):1019–30. 10.2165/00007256-200636120-00003. 17123326 10.2165/00007256-200636120-00003

[35] Barnett LM, Lai SK, Veldman SLC, Hardy LL, Cliff DP, Morgan PJ, Zask A, Lubans DR, Shultz SP, Ridgers ND, Rush E, Brown HL, Okely AD. Correlates of gross motor competence in children and adolescents: A systematic review and meta-analysis. Sports Med. 2016;46(11):1663–88. 10.1007/s40279-016-0495-z. 26894274 10.1007/s40279-016-0495-z PMC5055571

[36] Erhardt N, Martin-Rios C, Harkins J. Knowledge flow from the top: the importance of teamwork structure in team sports. Eur Sport Manag Q. 2014;14(4):375–96. 10.1080/16184742.2014.929159.

[37] Rodriguez-Ayllon M, Cadenas-Sánchez C, Estévez-López F, Muñoz NE, Mora-Gonzalez J, Migueles JH, Molina-García P, Henriksson H, Mena-Molina A, Martínez-Vizcaíno V, Catena A, Löf M, Erickson KI, Lubans DR, Ortega FB, Esteban-Cornejo I. Role of physical activity and sedentary behavior in the mental health of preschoolers, children and adolescents: A systematic review and meta-analysis. Sports Med. 2019;49(9):1383–410. 10.1007/s40279-019-01099-5. 30993594 10.1007/s40279-019-01099-5

[38] Fonseca APLM, de Azevedo CVM, Santos RMR. Sleep and health-related physical fitness in children and adolescents: a systematic review. Sleep Sci. 2021;14(4):357–65. 10.5935/1984-0063.20200125. 35087633 10.5935/1984-0063.20200125 PMC8776269

[39] Verrillo E, Bizzarri C, Cappa M, Bruni O, Pavone M, Ferri R, Cutrera R. Sleep characteristics in children with growth hormone deficiency. Neuroendocrinology. 2011;94(1):66–74. 10.1159/000326818. 21464567 10.1159/000326818

[40] Malhotra RK, Sleep. Recovery, and Performance in Sports. Neurol Clin. 2017;35(3):547–57. 10.1016/j.ncl.2017.03.002. 28673415 10.1016/j.ncl.2017.03.002

[41] Louca M, Short MA. The effect of one night’s sleep deprivation on adolescent neurobehavioral performance. Sleep. 2014;37(11):1799–807. 10.5665/sleep.4174. 25364075 10.5665/sleep.4174 PMC4196063

[42] Maquet P. The role of sleep in learning and memory. Science. 2001;294(5544):1048–52. 10.1126/science.1062856. 11691982 10.1126/science.1062856

[43] Kuna K, Szewczyk K, Gabryelska A, Białasiewicz P, Ditmer M, Strzelecki D, Sochal M. Potential role of sleep deficiency in inducing immune dysfunction. Biomedicines. 2022;10(9):2159. 10.3390/biomedicines10092159. 36140260 10.3390/biomedicines10092159 PMC9496201

[44] Ueno DT, Guerra PH, Christofoletti AEM, Bonolo A, Nakamura PM, Kokubun E. Mobile health apps to reduce sedentary behavior: a scoping review. Health Promot Int. 2022;37(2):daab124. 10.1093/heapro/daab124. 34392354 10.1093/heapro/daab124

[45] Gabel L, Macdonald HM, Nettlefold L, McKay HA. Physical activity, sedentary time, and bone strength from childhood to early adulthood: A mixed longitudinal HR-pQCT study. J Bone Min Res. 2017;32(7):1525–36. 10.1002/jbmr.3115. 10.1002/jbmr.3115 28326606

[46] Gianoudis J, Bailey CA, Daly RM. Associations between sedentary behaviour and body composition, muscle function and sarcopenia in community-dwelling older adults. Osteoporos Int. 2015;26(2):571–9. 10.1007/s00198-014-2895-y. 25245026 10.1007/s00198-014-2895-y

[47] Fraiwan M, Audat Z, Fraiwan L, Manasreh T. Using deep transfer learning to detect scoliosis and spondylolisthesis from x-ray images. PLoS ONE. 2022;17(5):e0267851. 10.1371/journal.pone.0267851. 35500000 10.1371/journal.pone.0267851 PMC9060368

[48] Cecchini M, Sassi F, Lauer JA, Lee YY, Guajardo-Barron V, Chisholm D. Tackling of unhealthy diets, physical inactivity, and obesity: health effects and cost-effectiveness. Lancet. 2010;376(9754):1775–84. 10.1016/S0140-6736(10)61514-0. 21074255 10.1016/S0140-6736(10)61514-0

[49] Hopkins SE, Orr E, Boyer BB, Thompson B. Culturally adapting an evidence-based intervention to promote a healthy diet and lifestyle for Yup’ik Alaska native communities. Int J Circumpolar Health. 2023;82(1):2159888. 10.1080/22423982.2022.2159888. 36544274 10.1080/22423982.2022.2159888 PMC9788688

[50] de la Peña JB, Ahsan HM, Botanas CJ, Dela Peña IJ, Woo T, Kim HJ, Cheong JH. Cigarette smoke exposure during adolescence but not adulthood induces anxiety-like behavior and locomotor stimulation in rats during withdrawal. Int J Dev Neurosci. 2016;55:49–55. 10.1016/j.ijdevneu.2016.09.007. 27647343 10.1016/j.ijdevneu.2016.09.007

[51] Dai X, Dharmage SC, Lowe AJ, Allen KJ, Thomas PS, Perret J, Waidyatillake N, Matheson MC, Svanes C, Welsh L, Abramson MJ, Lodge CJ. Early smoke exposure is associated with asthma and lung function deficits in adolescents. J Asthma. 2017;54(6):662–9. 10.1080/02770903.2016.1253730. 27791435 10.1080/02770903.2016.1253730

[52] Huttunen R, Heikkinen T, Syrjänen J. Smoking and the outcome of infection. J Intern Med. 2011;269(3):258–69. 10.1111/j.1365-2796.2010.02332.x. 21175903 10.1111/j.1365-2796.2010.02332.x

[53] Song H, Yang X, Yang W, Dai Y, Duan K, Jiang X, Huang G, Li M, Zhong G, Liu P, Chen J. Cigarettes smoking and e-cigarettes using among university students: a cross-section survey in Guangzhou, China, 2021. BMC Public Health. 2023;23(1):438. 10.1186/s12889-023-15350-2. 36882716 10.1186/s12889-023-15350-2 PMC9990220

[54] Niskanen L, Laaksonen DE, Nyyssönen K, Punnonen K, Valkonen VP, Fuentes R, Tuomainen TP, Salonen R, Salonen JT. Inflammation, abdominal obesity, and smoking as predictors of hypertension. Hypertension. 2004;44(6):859–65. 10.1161/01.HYP.0000146691.51307.84. 15492131 10.1161/01.HYP.0000146691.51307.84

[55] Dinas PC, Koutedakis Y, Flouris AD. Effects of active and passive tobacco cigarette smoking on heart rate variability. Int J Cardiol. 2013;163(2):109–15. 10.1016/j.ijcard.2011.10.140. 22100604 10.1016/j.ijcard.2011.10.140

[56] Afghani A, Xie B, Wiswell RA, Gong J, Li Y, Anderson Johnson C. Bone mass of Asian adolescents in China: influence of physical activity and smoking. Med Sci Sports Exerc. 2003;35(5):720–9. 10.1249/01.MSS.0000064940.76574.BD. 12750579 10.1249/01.MSS.0000064940.76574.BD

[57] Rom O, Kaisari S, Aizenbud D, Reznick AZ. Cigarette smoke and muscle catabolism in C2 myotubes. Mech Ageing Dev. 2013;134(1–2):24–34. 10.1016/j.mad.2012.11.004. 23262287 10.1016/j.mad.2012.11.004

[58] Tice AL, Laudato JA, Fadool DA, Gordon BS, Steiner JL. Acute binge alcohol alters whole body metabolism and the time-dependent expression of skeletal muscle-specific metabolic markers for multiple days in mice. Am J Physiol Endocrinol Metab. 2022;323(3):E215–30. 10.1152/ajpendo.00026.2022. 35793479 10.1152/ajpendo.00026.2022 PMC9423784

[59] Hyun J, Han J, Lee C, Yoon M, Jung Y. Pathophysiological aspects of alcohol metabolism in the liver. Int J Mol Sci. 2021;22(11):5717. 10.3390/ijms22115717. 34071962 10.3390/ijms22115717 PMC8197869

[60] Steiner JL, Lang CH. Dysregulation of skeletal muscle protein metabolism by alcohol. Am J Physiol Endocrinol Metab. 2015;308(9):E699–712. 10.1152/ajpendo.00006.2015. 25759394 10.1152/ajpendo.00006.2015 PMC4420901

[61] Ohira T, Tanigawa T, Tabata M, Imano H, Kitamura A, Kiyama M, Sato S, Okamura T, Cui R, Koike KA, Shimamoto T, Iso H. Effects of habitual alcohol intake on ambulatory blood pressure, heart rate, and its variability among Japanese men. Hypertension. 2009;53(1):13–9. 10.1161/HYPERTENSIONAHA.108.114835. 19029490 10.1161/HYPERTENSIONAHA.108.114835

[62] Battista K, Leatherdale ST. Estimating how extra calories from alcohol consumption are likely an overlooked contributor to youth obesity. Health Promot Chronic Dis Prev Can. 2017;37(6):194–200. 10.24095/hpcdp.37.6.03. 28614047 10.24095/hpcdp.37.6.03 PMC5650014

[63] Romeo J, Wärnberg J, Nova E, Díaz LE, Gómez-Martinez S, Marcos A. Moderate alcohol consumption and the immune system: A review. Br J Nutr. 2007;98(Suppl 1):S111–5. 10.1017/S0007114507838049. 17922947 10.1017/S0007114507838049

[64] Kärkkäinen U, Mustelin L, Raevuori A, Kaprio J, Keski-Rahkonen A. Do disordered eating behaviors have long-term health-related consequences? Eur Eat Disord Rev. 2018;26(1):22–8. 10.1002/erv.2568. 29160017 10.1002/erv.2568 PMC5732059

[65] Su Y, Yang J, Zhang G, Tao J, Lu Q, Hu D, Liu Z, Su Y, Xu H. Longitudinal study on association between sugar-sweetened beverages consumption and insomnia among college students in Yunnan Province. Chin J Sch Health. 2024;45(10):1451–4,1459. 10.16835/j.cnki.1000-9817.2024318.

[66] Gibney MJ, Barr SI, Bellisle F, Drewnowski A, Fagt S, Livingstone B, Masset G, Varela Moreiras G, Moreno LA, Smith J, Vieux F, Thielecke F, Hopkins S. Breakfast in human nutrition: The international breakfast research initiative. Nutrients. 2018;10(5):559. 10.3390/nu10050559. 29723985 10.3390/nu10050559 PMC5986439

[67] Vermorel M, Bitar A, Vernet J, Verdier E, Coudert J. The extent to which breakfast covers the morning energy expenditure of adolescents with varying levels of physical activity. Eur J Clin Nutr. 2003;57(2):310–15. 10.1038/sj.ejcn.1601546. 12571665 10.1038/sj.ejcn.1601546

[68] Mogre V, Atibilla J, Kandoh B. Association between breakfast skipping and adiposity status among civil servants in the Tamale metropolis. J Biomed Sci. 2013;2(3)1–7. 10.4314/jmbs.v2i3.1.

[69] Khongrangjem T, Dsouza SM, Prabhu P, Dhange VB, Pari V, Ahirwar SK, Sumit K. A study to assess the knowledge and practice of fast food consumption among pre-university students in Udupi Taluk, Karnataka, India. Clin Epidemiol Global Health. 2018;6(4):172–5. 10.1016/j.cegh.2017.11.003.

[70] Beal T, Morris SS, Tumilowicz A. Global patterns of adolescent fruit, vegetable, carbonated soft drink, and fast-food consumption: A meta-analysis of global school-based student health surveys. Food Nutr Bull. 2019;40(4):444–59. 10.1177/0379572119848287. 31617415 10.1177/0379572119848287

[71] Alsabieh M, Alqahtani M, Altamimi A, Albasha A, Alsulaiman A, Alkhamshi A, Habib SS, Bashir S. Fast food consumption and its associations with heart rate, blood pressure, cognitive function and quality of life. Pilot study. Heliyon. 2019;5(5):e01566. 10.1016/j.heliyon.2019.e01566. 31193345 10.1016/j.heliyon.2019.e01566 PMC6526229

