# Supplementary material for: Physical fitness status and influencing factors among college students aged 19 ~ 22 in Shandong province, China: a cross-sectional study
Source: BMC Public Health. 2025 Jun 5;25:2105. doi: 10.1186/s12889-025-22350-x (PMC12139287; doi:10.1186/s12889-025-22350-x)
Supplement: Supplementary file 1 — Supplementary Material 1 [file 12889_2025_22350_MOESM1_ESM.docx]

Supplementary Table 1 Questionnaire

Dear participants,

Welcome to our study, which aims to understand the current status of physical fitness and its influencing factors among 19 ~ 22-year-old college students. We kindly ask you to take a few moments to answer the following questions. Please note that this is an entirely anonymous survey, and we will not collect any information that can identify you personally. There are no right or wrong answers, and we encourage you to respond based on your true experiences and circumstances. Your participation is greatly appreciated.

(1) Gender

⓪ Male ① Female

(2) Age ___

(3) Household registration

⓪ Rural household registration ① Urban household registration

(4) Family annual income (yuan)

①≤ 100000 ② 100001 **~** 200000 ③ 200001 **~** 300000 ④ ≥ 300000

(5) Paternal education level

① Junior high school or below ② Senior high school ③ Above senior high school

(6) Maternal education level

① Junior high school or below ② Senior high school ③ Above senior high school

(7) Whether parents like physical exercise

① Neither side of the parents likes ② One side of the parents likes

③ Both sides of the parents like

(8) Whether parents support your participation in physical exercise

⓪ Nonsupporting ① Supporting

(9) Number of physical exercise sessions per week

① < 3 ② 3 ~ 5 ③ > 5

(10) Duration of each physical exercise session (hour)

① < 0.5 ② 0.5 ~ 1 ③ > 1

(11) Intensity of physical exercise

① Low intensity ② Moderate intensity ③ High intensity

(12) Sleep duration per day (hour)

① < 6 ② 6 ~ 8 ③ > 8

(13) Screen duration per day (hour)

① < 1 ② 1 ~ 3 ③ > 3

(14) Times of smoking per week

① Never ② 1 ~ 4 ③ > 4

(15) Times of drinking per week

① < 2 ② 2 ~ 4 ③ > 4

(16) Times of breakfast intake per week

① Never ② 1 ~ 2 ③ 3 ~ 6 ④ Everyday

(17) Times of meat intake per week

① < 3 ② 3 ~ 4 ③ > 4

(18) Times of vegetable intake per week

① < 4 ② 4 ~ 5 ③ > 5

(19) Times of fruit intake per week

① < 4 ② 4 ~ 5 ③ > 5

(20) Times of eggs intake per week

① < 4 ② 4 ~ 5 ③ > 5

(21) Times of milk intake per week

① < 4 ② 4 ~ 5 ③ > 5

(22) Times of fast food consumption per week

① < 2 ② 2 ~ 3 ③ > 3
